# Enhanced Virulence of *Candida albicans* by *Staphylococcus aureus*: Evidence in Clinical Bloodstream Infections and Infected Zebrafish Embryos

**DOI:** 10.3390/jof7121099

**Published:** 2021-12-20

**Authors:** Yen-Mu Wu, Po-Yen Huang, Yi-Chuan Cheng, Chih-Hua Lee, Meng-Chieh Hsu, Jang-Jih Lu, Shao-Hung Wang

**Affiliations:** 1 Department of Internal Medicine, Division of Infectious Diseases, Chang Gung Memorial Hospital at Linkou Medical Center, Taoyuan City 333, Taiwan; yenmuwu@gmail.com (Y.-M.W.); pyhuang@gmail.com (P.-Y.H.); 2 Graduate Institute of Clinical Medical Sciences, College of Medicine, Chang Gung University, Taoyuan City 333, Taiwan; 3 Graduate Institute of Biomedical Sciences, College of Medicine, Chang Gung University, Taoyuan City 333, Taiwan; yccheng@mail.cgu.edu.tw; 4 Neuroscience Research Center, Chang Gung Memorial Hospital at Linkou Medical Center, Taoyuan City 333, Taiwan; 5 Department of Laboratory Medicine, Chang-Gung Memorial Hospital at Linkou Medical Center, Taoyuan City 333, Taiwan; josh60708@cgmh.org.tw; 6 Department of Microbiology, Immunology and Biopharmaceuticals, National Chiayi University, Chiayi City 600, Taiwan; a23235630@gmail.com; 7 Department of Medical Biotechnology and Laboratory Science, Chang Gung University, Taoyuan City 333, Taiwan; 8 Department of Medicine, College of Medicine, Chang Gung University, Taoyuan City 333, Taiwan

**Keywords:** mixed BSI, virulence, co-biofilm, *Candida albicans*, *Staphylococcus aureus*, hyphal morphogenesis, zebrafish embryo infection

## Abstract

Coinfection with *Candida* and *Staphylococcus* results in higher mortality in animal studies. However, the pathogenesis and interplay between *C. albicans* and *S. aureus* in bloodstream infections (BSIs) is unclear. This study determines the clinical features and outcomes of mixed *C. albicans*/*S. aureus* (CA/SA) BSIs and biofilm formation on pathogenesis during coinfection. Demographics and outcomes for mixed BSIs and monomicrobial candidemia were compared. Compared to 115 monomicrobial *C. albicans* BSIs, 22 patients with mixed CA/SA BSIs exhibited a significantly higher mortality rate and shorter survival time. In vitro and in vivo biofilm analysis showed that *C. albicans* accounted for the main biofilm architecture, and *S. aureus* increased its amount. Antibiotic tolerance in *S. aureus*, which adhered to *Candida* hyphae observed by scanning electron microscope, was demonstrated by the presence of wild-type *C. albicans* co-biofilm. Upregulation in exotoxin genes of *S. aureus* was evidenced by quantitative RT-PCR when a co-biofilm was formed with *C. albicans*. Mixed CA/SA BSIs result in a higher mortality rate in patients and in vivo surrogate models experiments. This study demonstrates that the virulence enhancement of *C. albicans* and *S. aureus* during co-biofilm formation contributes to the high mortality rate.

## 1. Introduction

The global SENTRY Antimicrobial Surveillance Program from 2012–2017 demonstrated that *Staphylococcus aureus*, which was the most prevalent bacterial pathogen, caused 22.5% of bloodstream infections (BSIs), and *Candida* species caused 3.1% [1], with increasing prevalence during the last decade [2]. Mixed BSIs with bacteria and *Candida* results in a more severe prognosis and are associated with higher morbidity and mortality than monomicrobial or polybacterial BSIs [3,4,5].

*S. aureus* and *C. albicans* are both important lethal pathogens in nosocomial BSIs, and there is an increase in the incidence of mixed BSI, so the risk factors and prognosis of mixed *C. albicans*/*S. aureus* (CA/SA) BSIs in the population and the interplay between *C. albicans* and *S. aureus* in host pathogenesis are worthy of investigation.

Coinfection models involving *Candida* and *Staphylococcus* have been used in several animal studies [6,7,8,9,10,11,12]. These studies all show that mixed-infection with *Candida* and *Staphylococcus* increases mortality of the infected hosts, and the interplay between the bacterium and the fungus significantly increases the bacterial burden or virulence on hosts. It has been demonstrated that a biofilm of *Candida* protects bacteria from host defensiveness, and each microbial secretory factor affects their virulence [13,14].

Embryos of zebrafish are commonly used as a surrogate infection host and are used as a model to determine the biofilm activity of *C. albicans* in vivo. This model allows antifungal screening and genetic studies with the advantages of conserved immunity, prolific fecundity, and cost-effectiveness [15,16,17,18]. In order to determine the interplay between *C. albicans* and *S. aureus* in infected hosts without immune interference, a zebrafish embryo infection assay was used for this study.

## 2. Materials and Methods

### 2.1. Study Design and Definition

This retrospective study was conducted from 2003 to 2015 at Chang Gung Memorial Hospital at Linkou (CGMHL) and was approved by the institutional review board of the hospital (201701182B0 and 202101345B0). Adult hospitalized patients (≥18 years old) were recruited. Mixed CA/SA BSI cases were defined by a positive blood culture for *C. albicans* with *S. aureus* growing concomitantly from the same set. Data from adult patients who had monomicrobial *C. albicans* BSIs in 2010 were used for comparison.

### 2.2. Clinical Characteristics and Outcomes

Data for demographics, comorbidities, risk factors, and clinical outcomes were recorded. Comorbidities included heart failure, chronic lung diseases, diabetes mellitus, hepatic dysfunction (a serum total bilirubin level ≥ 2.0 mg/dL or liver cirrhosis), renal insufficiency (a serum creatinine level ≥ 2.0 mg/dL or a requirement for hemodialysis), hematological malignancies, and solid tumors. The risk factors collected within 30 days before the BSIs included abdominal surgery, central venous catheterization, parenteral nutrition, immunosuppressant administration (prednisolone ≥ 20 mg/day for more than 3 weeks, chemotherapeutic and other immunosuppressive drugs), and neutropenia (an absolute neutrophil count < 500 cells/mL). Severity of illness was assessed at the onset of BSIs using a sequential organ failure assessment (SOFA) score [19]. Clinical outcomes were assessed in terms of mortality, median survival days after BSIs, and length of stay in hospital. The clinical characteristics and outcomes for *C. albicans* candidemia were described in a previous study by the authors [20].

### 2.3. Microbes and Fish Strains

To evaluate the virulence enhancement of *C. albicans* biofilm by *S. aureus*, a virulent laboratory strain SC5314 and its biofilm-defective mutant HLC54 were chosen as well as a hemolysin-producing *S. aureus* ATCC 29213, which is the most commonly used strain in the clinical laboratory. *C. albicans* HLC54 (*cph1*/*cph1 efg1*/*efg1*) is a hyphae-defective mutant derived from virulent parental SC5314 [21]. A clinical isolate P004 with low biofilm was also used as the fungal pathogen [17]. All yeasts are caspofungin susceptible. A methicillin-susceptible *S. aureus* ATCC 29213 was used as the bacterial pathogen, which is vancomycin susceptible [22]. Wild-type zebrafish (*Danio rerio*) AB line (Zebrafish International Resource Center, Eugene, OR, USA), aged approximately 8–15 months, were maintained at 28 °C in a 10-h dark 14-h light cycle to collect fertilized eggs, which served as surrogate hosts. All experiments were performed in accordance with standard guidelines for zebrafish studies [23].

### 2.4. In Vitro Biofilm Analysis

3-Morpholinopropane sulfonic acid (MOPS)-buffered RPMI-1640 (pH7.2) containing 10% fetal calf serum and 1.25% *N*-acetyl-d-glucosamine was used as a biofilm formation medium. For the metabolic reducing dye assay, overnight-cultivated microbes were refreshed for 4 h, 10^5^ cfu of the cultures were seeded into microplates, and then, biofilm that was cultured at 37 °C for 4-h or 24-h was measured using a CCK-8 kit (cat. 96992, Sigma-Aldrich, St. Louis, MO, USA). For the other biofilm quantification method using safranin, sterile 13-mm hydrophilic filters (cat. AAWP01300, Millipore, Bedford, MA, USA) were used as an adherent surface, and biofilm-stained Gram’s safranin was dissolved using 30% acetic acid and measured using OD_530_.

### 2.5. Zebrafish Embryo Infection Assay

The infection assay follows the method previously described [17]. Embryos were co-incubated with 5 × 10^5^ yeast/mL *C. albicans* and/or 2 × 10^7^ cfu/mL *S. aureus* in RPMI-1640 medium (Thermo Fisher Scientific, Waltham, MA, USA) at 120 rpm 30 °C for 4 h. After 4-h co-incubation, the infected embryos were washed thrice with egg water (0.03% sea salt) to remove non-adherent microbes and then transferred into fresh egg water supplemented with 0.5% YPD broth and were grown at 30 °C for 24 h. The survival rate was calculated as the percentage of the death by monitoring the heartbeat of embryos. All experiments were approved by the Biosafety Committee (No. 00417-2020092830581) and IACUC (CGU109-109) of the hospital and were conducted in a BSL-2 laboratory.

### 2.6. Microbial Enumeration

After 4-h adhesion, the microbial infected chorions were incubated for 20 h to allow biofilm maturation and were treated with antimicrobials (80 μg/mL vancomycin or 0.5 μg/mL caspofungin). The chorions were washed and transferred to microtubes and ground using a pestle. The resulting mixtures were colony counted for microbes on suitable agar plates with vancomycin (4 μg/mL) or caspofungin (4 μg/mL).

### 2.7. Scanning Electron Microscope (SEM)

Samples were washed and harvested after 24-h incubation and fixed in a mixture of 3% gutaraldehyde and 2% paraformaldehyde that was buffered to pH 7.4 using 0.1 M cacodylate buffer. Samples were washed with cacodylate buffer and treated with 1% osmium tetroxide. The cell pellets were washed thoroughly with 0.1 M cacodylate buffer and dehydrated gradually with ethanol until only 100% ethanol remained. After critical-point drying and mounting on an SEM stub, the samples were sputter-coated with a thin layer of gold, and images were recorded at an appropriate accelerating voltage.

### 2.8. Gene Expression Assays

The biofilm mass was dissolved and ground in REzol C&T reagent (Protech Technology, Taipei, Taiwan) and kept frozen before RNA extraction. To determine the expression of *S. aureus* toxin genes in the biofilm, total RNA extracted with MagNA Pure Compact RNA Isolation Kit (Roche Applied Science, Indianapolis, IN, USA) was two-step reverse-transcribed (High Capacity cDNA Reverse Transcription Kit, Applied Biosystems, Foster City, CA, USA) to cDNA and analyzed by quantitative PCR run in ABI 7900 HT Real-Time PCR System: 10 min at 95 °C and 40 cycles of 15 sec at 95 °C, 1 min at 60 °C using 2× Gene Expression Master Mix (Applied Biosystems, Foster City, CA, USA). The primers (Mission Biotech, Taipei, Taiwan) and TaqMan probes (Thermo Fisher Scientific, Waltham, MA, USA) corresponding to *S. aureus* housekeeping gene *rrsA* and hemolysin genes *hla* and *hlgB* are listed in Table S1. The change in the expression was calculated using the comparative CT method, with *rrsA* as an endogenous control.

### 2.9. Statistical Analysis

Statistical analysis used SPSS 22.0 software (IBM, Armonk, NY, USA). Continuous variables are presented as mean ± standard deviation (SD) if the data are normally distributed and as a median and interquartile range (IQR) if the data are non-normally distributed. Categorical variables were compared using Pearson’s chi-square test or Fisher’s exact test and continuous variables were compared using a Student’s *t*-test or a Mann–Whitney U test. Variables with a two-tailed *p*-value < 0.01 were included in a binary logistic regression model for a multivariate analysis. All tests were two-tailed, and a *p*-value of <0.05 represents statistical significance.

## 3. Results

### 3.1. Clinical Characterization of Mixed C. albicans/S. aureus BSIs

A total of 22 patients with mixed CA/SA BSIs from 2003–2015 and 115 monomicrobial *C. albicans* BSI patients in 2010 at CGMHL were included in this study. The demographics, clinical characteristics, risk factors, and outcomes were compared. More patients in mixed CA/SA BSI group exhibited renal insufficiency (59.1% vs. 27.0%, *p* = 0.003). Unlike *C. albicans* candidemia, mixed CA/SA BSI cases were associated with a higher SOFA score (10.6 vs. 7.0, *p* = 0.003), higher 14-/30-day mortality rate (14-day: 77.3% vs. 40.0%, *p* = 0.002; 30-day: 81.8% vs. 53.9%, *p* = 0.018), and shorter median survival days (4.5 vs. 25.0, *p* = 0.001) (Table 1). Within 22 mixed CA/SA BSIs, five patients died within 24 h after blood cultures were obtained. Of the remaining 17 patients, intravascular catheters were removed in 13 (76.5%), and follow-up blood cultures were performed in five (29.4%), yielding no growth of *C. albicans* or *S. aureus*.

Patients who did not survive more than 14 days were correlated with liver dysfunction (39.7% vs. 13.5%, *p* < 0.001), renal insufficiency (52.4% vs. 14.9%, *p* < 0.001), higher SOFA score (11 vs. 4, *p* < 0.001), and more mixed CA/SA BSIs (27.0% vs. 6.8%, *p* = 0.001) (Table 2). Multivariate analysis showed that 14-day mortality was positively associated with a high SOFA score (OR 1.13, 95% CI 1.03–1.24) and mixed CA/SA BSIs (OR 3.47, 95% CI 1.05–11.50) (Table 3).

### 3.2. Co-Biofilm of C. albicans and S. aureus In Vitro

**Figure 1:**
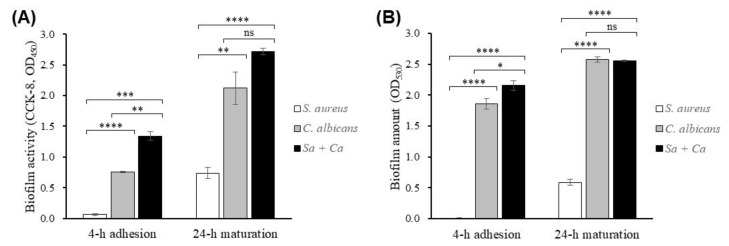
Analysis of in vitro microbial biofilm with *C. albicans* and *S. aureus*. Using microplates or hydrophilic membrane filters as an adherent surface, 10^5^ cfu of *C. albicans* and/or *S. aureus* strains were seeded for 4-h adhesion and 24-h maturation of biofilms at 37 °C and analyzed using a CCK-8 kit (**A**) or Gram’s safranin dye staining (**B**). Note: * *p* < 0.05, ** *p* < 0.01, *** *p* < 0.001, **** *p* < 0.0001, ns = not significant.

The in vitro biofilm activity assay was performed using a metabolic reducing dye and Gram’s safranin staining. The results for the two biofilm assays showed that there was significantly higher activity in the mixed-infection group than in the monomicrobial groups (Figure 1). The difference of biofilm in 4-h adhesion between the mixed-infection group and others was more significant than that for the 24-h maturation, and the results showed that *C. albicans* accounted for most biofilm structure in terms of both the activity and the amount of biofilm. β-glucan in culture supernatants of *C. albicans* has been shown to potentiate drug resistance of *S. aureus* [24]. Biofilm enhancement in microbial supernatants was also studied, and a significant increase in *S. aureus* biofilm with *C. albicans* supernatants was observed in terms of the activity and the amount (Figure S1).

### 3.3. Enhancement of Virulence of C. albicans Biofilm by S. aureus on Zebrafish Embryos

**Figure 2:**
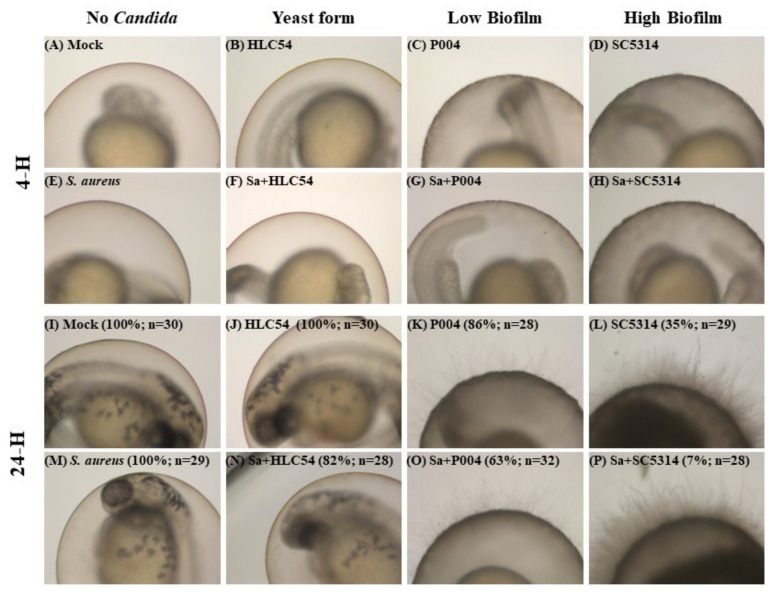
Analysis of in vivo microbial biofilms on zebrafish chorions with *C. albicans* and *S. aureus*. After 4-h adhesion, bound microorganisms, including *S. aureus* ATCC 29213, *C. albicans* hyphae-defective mutant HLC54, low biofilm clinical strain P004, and wild-type SC5314 strains, were cultivated at 30 °C for 24 h to form mature biofilms (**A**–**P**). Photographs were taken during the 4-h adhesion and 24-h maturation periods. The survival rate for infected embryos was measured and is shown in parentheses with the number (*n*) of eggs that was used for each experiment. Around 30 embryos were used for each test. The heart-rate of fish embryos was measured to determine the survival rate.

Within 4-h incubation, the microbial loads for *S. aureus* and *C. albicans* SC5314 grown on the surface of egg chorion were, respectively, calculated to be 1.5 × 10^6^ cfu and 3.5 × 10^6^ cfu per egg (data not shown). After 24-h development, no matter whether *S. aureus* was present, heavy and thick biofilms were observed on chorions of the SC5314 group and the low-biofilm P004 group (Figure 2K,L,O,P), but there was no obvious biofilm observed in neither *S.* *aureus* nor hyphae-defective HLC54-infected groups (Figure 2J,M,N).

Co-cultivation of *C. albicans* significantly increased the bacterial load in the co-biofilm on eggs from 2.3 × 10^7^ to 6.9 × 10^7^ in the SC5314 group and 2.6 × 10^8^ in the HLC54 group (Table 4). Significantly fewer embryos in the co-infected groups survived and this reduction was much greater for wild-type SC5314: from 35% to 7% (Figure 2L,P).

### 3.4. Protection of S. aureus against Antibiotics in a Co-Biofilm with Hyphal C. albicans

**Figure 3:**
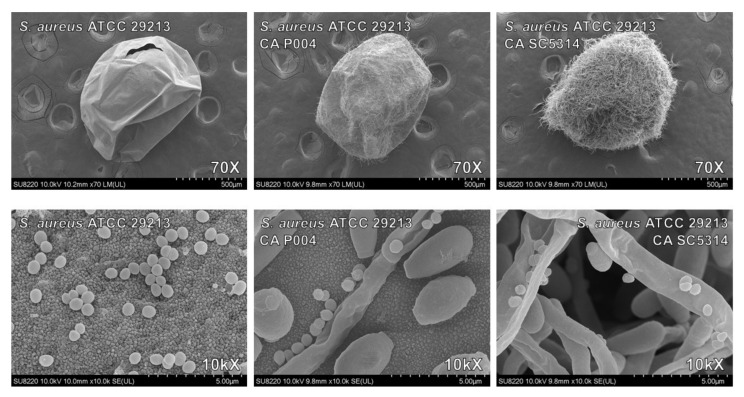
Scanning electron microscope graphs of biofilm morphology on fish embryo chorions. After 4-h adhesion and 24-h maturation, fish embryos that were infected with *S. aureus* ATCC 29213 only or that were co-infected with a low biofilm clinical strain P004 or wild-type SC5314 strains were used for SEM.

**Figure 4:**
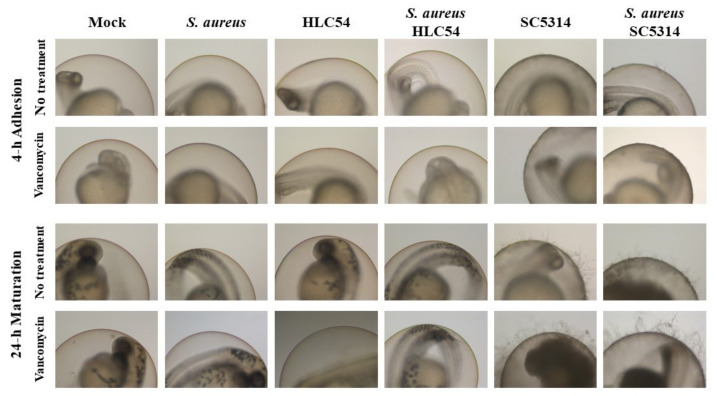
The morphology of mixed biofilm that are treated with *S. aureus* and *C. albicans* under vancomycin. After 4-h adhesion and 24-h maturation, fish embryos that were infected with *S. aureus* ATCC 29213, a hyphae-defective mutant HLC54, or wild-type SC5314 strains individually or mixed infected were photographed using a stereotactic microscope. Rows 2 and 4 share the same preparation of infected embryos but involve supplementation of 80 μg/mL vancomycin.

The *S. aureus* loads after vancomycin treatment were significantly increased in co-biofilm with wild-type SC5314, while the protection declined when formed with a hyphae-defective HLC54 (Table 4). However, no obvious protective effect was observed in the co-biofilms for *C. albicans* against caspofungin, either SC5314 or HLC54 (data not shown). The scanning electron microscope (SEM) results showed that *S. aureus* almost adhered to *C. albicans* hyphae (Figure 3), but yeast cells did not express molecules for intimate interaction with *S. aureus* to shield from drug damage. There was no obvious change in the biofilm morphology for co-infected embryos that were treated with vancomycin, which eliminated non-hyphal protected *S. aureus* (Figure 4).

### 3.5. Changes in S. aureus Toxin Genes in A Co-Biofilm

**Figure 5:**
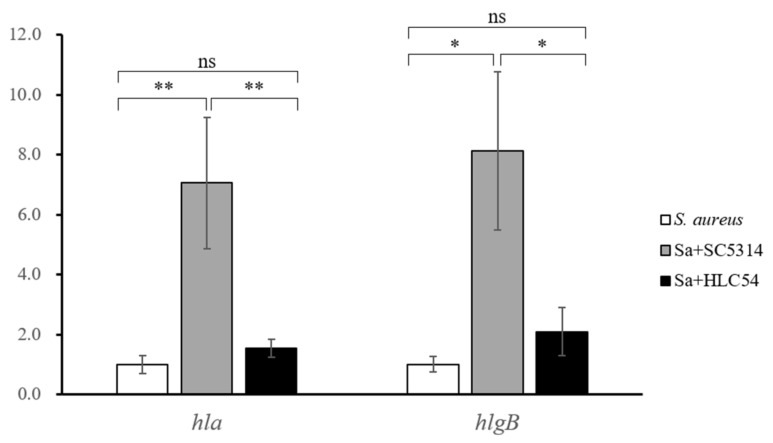
The upregulation of *S. aureus* toxin genes *hla* and *hlgB* in a mixed biofilm that is formed by *S. aureus* and *C. albicans* SC5314. After 24-h maturation of biofilms on filter membranes, the in vitro biofilms that were formed with *S. aureus* only (SA, white bars), *S. aureus* co-cultivated with *C. albicans* SC5314 (SA + SC5314, gray bars), or with hyphae-defective mutant HLC54 (SA + HLC54, black bars) were washed twice, and total RNA was isolated and reverse transcribed to determine gene expression. Note: * *p* < 0.05, ** *p* < 0.01, ns = not significant.

*S. aureus* toxin genes were augmented by *C. albicans* in murine infection models, including *hla* (alpha hemolysin) and *hlgB* (gamma hemolysin) [25]. If *S. aureus* formed co-biofilms with *C. albicans* on a filter membrane after 24-h maturation, there was a significant increase in expression of *hla* (7.06 ± 2.19 fold increase) and *hlgB* (8.12 ± 2.64 fold increase) in SC5314 group but not in hyphae-defective HLC54 group (*hla*: 1.54 ± 0.30 and *hlgB*: 2.09 ± 0.81) (Figure 5).

## 4. Discussion

This study demonstrates that the clinical outcome strongly supports the results for an in vitro enhanced virulence for candidemia by *S. aureus*. Within the monomicrobial *C. albicans* BSI group, more patients were found with parenteral nutrition and immunosuppressants, so this population was more immunocompromised. However, in comparison with this group, 22 mixed CA/SA BSI patients, enrolled from 264 mixed CA/bacterial BSIs (2003–2015), were associated with a higher SOFA score, increased 14-day and 30-day mortalities, and shorter median survival days. This demonstrates that *S. aureus* increases the virulence of *C. albicans* in BSI patients.

Zhong et al. reported that patients with mixed *Candida albicans*/bacterial bloodstream infections in China undergo mechanical ventilation for longer and stay longer in the ICU than patients with monomicrobial *C. albicans* BSIs, but there is no difference in 28-/60-day mortality [5]. The difference in mortality for mixed BSIs in different studies may be due to different bacterial pathogens. *Staphylococcus* is associated with higher mortality for animal hosts [6,7,8,9,10,11], but *Enterococcus* spp. negatively affects each other’s virulence [26,27,28].

In terms of comorbidity, there is a higher incidence of renal insufficiency in mixed BSI group (Table 1). Patients who did not survive for 14 days had a higher prevalence of liver dysfunction and renal insufficiency and had almost four-times higher incidence of mixed CA/SA BSIs (Table 2). The authors demonstrated previously that a higher prevalence of renal insufficiency was associated with high-biofilm *C. albicans* isolates [17]. Herein, in vivo zebrafish embryo infection assay exhibited embryos co-infected with *S. aureus* and high-biofilm *C. albicans* survived less. Therefore, renal insufficiency is closely associated with mixed CA/SA BSI. In a 12-year period autopsies review, the most frequently involved deep parenchymal organ in candidiasis was the kidney [29]. Shin et al. reported that severe renal failure plays a major role in lethality for mice infected with *C. albicans* [30]. In an intravenous mouse infection model, kidneys were demonstrated to be the main target of *C. albicans*, though the upregulation of hyphae-associated genes was measured in the liver without visible hyphal invasion [31].

Our study results show that *S. aureus* significantly increases biofilm formation of *C. albicans* in the in vitro abiotic assay and in vivo surrogate zebrafish model. The increased biofilm of *C. albicans* and enhanced virulence of *S. aureus* are the reason for higher mortality in mixed BSIs. For the biofilm model with zebrafish eggs, only wild-type *C. albicans* provides *S. aureus* with protection against vancomycin rather than hyphae-defective mutant (HLC54). SEM images show that there is tight contact between the hyphae of *C. albicans* and *S. aureus* in a thick and multilayer biofilm, in which there is a matrix that is composed of an entangled polymeric substance during maturation that shields against antimicrobials.

This study shows that the SOFA score was significant higher within mixed CA/SA BSI patients, so interaction between these two pathogens probably exacerbates tissue damage and erosion during hyphal invasion. Our embryo infection result suggested *C. albicans* co-biofilm protects *S. aureus* against antimicrobials, and the major toxin genes of *S. aureus* are significantly upregulated, including *hla* and *hlgB*, resulting in high mortality for *C. albicans* SC5314-infected embryos. An upregulation of virulence factors in *S. aureus*, including *hla*, *hlgB*, enterotoxin family protein, staphylocoagulase, staphylococcal protein A, nucleases, intercellular adhesion proteins, fibronectin-binding proteins, drug-resistant genes *glmU*, *murC,* and *murD*, and penicillin-binding proteins, was demonstrated by Hu et al. using cutaneous abscess and peritonitis murine models [25]. The increase in toxin production, especially exterotoxins, damages tissues and induces severe inflammation, which can cause multi-organ failure.

The study has some limitations. First, we acknowledge that insufficient information about follow-up blood cultures to confirm pathogen clearance was obtained in CA/SA BSI patients in this single-center retrospective study. However, these were critically ill patients with high mortality, and 12 of them (12/22 = 54.5%) died within seven days after mixed CA/SA BSIs occurred. Second, the zebrafish has recently become an extremely powerful model organism in the context of *Candida* infections and host-pathogen interactions [32]. However, the drawback of the model is that the embryos were collected and grown at 30 °C, which does not allow accurate mimicking of human infection.

## 5. Conclusions

Mixed CA/SA BSIs are associated with high morbidity and mortality in our hospital. The zebrafish embryo in vitro infection assay exhibits antibiotic tolerance in *S. aureus* if it attaches to the hyphae of *C. albicans*. Using in vitro assays, the production of *S. aureus* toxins in *Candida* co-biofilm possibly involves pathogenesis.

## Figures and Tables

**Table 1:** Comparison of clinical features and outcomes for patients in mixed CA/SA BSI and *C. albicans* candidemia groups.

Variables	Mixed CA/SA BSI	*C. albicans* Candidemia	Univariate
	***N* = 22**	***N* = 115**	***p*-Value**
Age (SD), years	73.1 (15.0)	66.5 (14.5)	0.053
Sex, male	7 (31.8)	64 (55.7)	0.061
**Comorbidities**			
Cardiovascular disease	4 (18.2)	16 (13.9)	0.530
Chronic lung disease	7 (31.8)	17 (14.8)	0.068
Diabetes mellitus	9 (40.9)	37 (32.2)	0.427
Liver dysfunction	6 (27.3)	29 (25.2)	0.796
Renal insufficiency	13 (59.1)	31 (27.0)	0.003
Hematological malignancy	1 (4.5)	6 (5.2)	1.000
Solid tumor	3 (13.6)	64 (55.7)	0.000
**Risk factors ^1^**			
Abdominal surgery	3 (13.6)	19 (16.5)	1.000
Intravascular catheter	20 (90.9)	107 (93.0)	0.663
Parenteral nutrition	4 (18.2)	60 (52.2)	0.004
Immunosuppressants	2 (9.1)	38 (33.0)	0.023
Neutropenia ^2^	1 (4.5)	6 (5.2)	1.000
**Clinical condition**			
SOFA score (SD)	10.6 (6.7)	7.0 (5.7)	0.003
LOS before BSIs (SD), days	48.5 (50.7)	34.2 (29.6)	0.309
**Clinical outcomes**			
14-day mortality	17 (77.3)	46 (40.0)	0.002
30-day mortality	18 (81.8)	62 (53.9)	0.018
Median survival days (IQR)	4.5 (1.75–11.00)	25.0 (16.8–33.2)	0.001
LOS (SD), days	64.1 (51.5)	58.8 (42.4)	0.606

Note: Categorical data are presented as no. (%) of subject. Mean (standard deviation (SD)) and median (interquartile range (IQR)) are, respectively, used to describe normally and non-normally distributed data. CA, *Candida albicans*; SA, *Staphylococcus aureus*; BSI, bloodstream infection; LOS, length of stay; SOFA, sequential organ failure assessment. ^1^ Risk factors were evaluated within 30 days before BSI occurrence. ^2^ An absolute neutrophil count <500 cells/mL.

**Table 2:** A comparison of the variables for the 14-day survival and non-survival groups.

Variables	14-D Mortality	14-D Non-Mortality	Univariate
	***N* = 63**	***N* = 74**	***p*-Value**
**Comorbidities**			
Cardiovascular disease	10 (15.9)	10 (13.5)	0.697
Chronic lung disease	10 (15.9)	14 (18.9)	0.640
Diabetes mellitus	24 (38.1)	22 (29.7)	0.301
Liver dysfunction	25 (39.7)	10 (13.5)	0.000
Renal insufficiency	33 (52.4)	11 (14.9)	0.000
Hematological malignancy	4 (6.3)	3 (4.1)	0.703
Solid tumor	25 (39.7)	42 (56.8)	0.046
**Clinical scenarios**			
Abdominal surgery	11 (17.5)	11 (14.9)	0.680
Intravascular catheter	60 (95.2)	67 (90.5)	0.342
Parenteral nutrition	30 (47.6)	34 (45.9)	0.845
Immunosuppressants	18 (28.6)	22 (29.7)	0.882
Neutropenia	2 (3.2)	5 (6.8)	0.452
SOFA score (IQR)	11 (4–15)	4 (1–7)	0.000
Mixed CA/SA BSI	17 (27.0)	5 (6.8)	0.001
LOS before BSI (SD), days	43.9 (40.4)	34.2 (29.7)	0.110

**Table 3:** Multivariate analysis of high variance factors that are associated with 14-day mortality for patients with BSIs.

Variables	14-D Mortality	14-D Non-Mortality	Multivariate	
	***N* = 63**	***N* = 74**	**OR (95% CI)**	***p*-Value**
Liver dysfunction	25 (39.7)	10 (13.5)	1.96 (0.70–5.48)	0.199
Renal insufficiency	33 (52.4)	11 (14.9)	2.18 (0.80–5.91)	0.126
SOFA score (IQR)	11 (4–15)	4 (1–7)	1.13 (1.03–1.24)	0.009
Mixed CA/SA BSI	17 (27.0)	5 (6.8)	3.47 (1.05–11.50)	0.041

**Table 4:** Protective strength of *S. aureus* from antibiotic assault due to *C. albicans* in the co-biofilm.

Treat	Infection Microorganisms	*S. aureus* Load (cfu/egg)
No Vancomycin	Mock infection	0
	*C. albicans* HLC54	0
	*C. albicans* SC5314	0
	*S. aureus*	2.3 × 10^7^
	*S. aureus* + HLC54	2.6 × 10^8^
	*S. aureus* + SC5314	6.9 × 10^7^
Vancomycin	*S. aureus*	0
	*S. aureus* + HLC54	0
	*S. aureus* + SC5314	3.0 × 10^7^

## Supplementary Materials

The following are available online at https://www.mdpi.com/article/10.3390/jof7121099/s1, Table S1: The primer sets and TaqMan probes for the gene expression assay; Figure S1: The enhancement of *S. aureus* biofilm using culture supernatants of *C. albicans*.
