# Supplementary material for: Enhanced Virulence of Candida albicans by Staphylococcus aureus: Evidence in Clinical Bloodstream Infections and Infected Zebrafish Embryos
Source: J Fungi (Basel). 2021 Dec 20;7(12):1099. doi: 10.3390/jof7121099 (PMC8706905; doi:10.3390/jof7121099)
Supplement: Supplementary file 1 [file jof-07-01099-s001.zip › jof-1494002-supplementary.pdf]

Journal of
Fungi

Table S1. The primer sets and TaqMan probes for the gene expression assay [1].

Gene Targeted	Primers/Probes	Nucleotide Sequence (5′ - 3′)
rrsA	*rrsA_For*	GCCCCTTAGTGCTGCAGCTA
	rrsA_Rev	AGTTTCAACCTTGCGGTCGTA
	rrsA_Probe	FAM-CGCATTAAGCACTCCGCCTGGG-MGB
hlgB	*hlgB_For*	CTTGCCCCTCTTGCCAATC
	hlgB_Rev	TTTGATAATTTCTATATCGCTTCCTTTACC
	hlgB_Probe	FAM-AAATGCTAAAGCTGCCAACGATACTGAAGACAT-MGB
hla	*hla_For*	CAACAACACTATTGCTAGGTTCCATATT
	hla_Rev	CCTGTTTTTACTGTAGTATTGCTTCCA
	hla_Probe	FAM-ATGAATCCTGTCGCTAATGCCGCAGA-MGB

Reference:

1. Loughman, J.A.; Fritz, S.A.; Storch, G.A.; Hunstad, D.A. Virulence gene expression in human community-acquired *Staphylococcus aureus* infection. *J Infect Dis* **2009**, *199*, 294-301, doi:10.1086/595982.

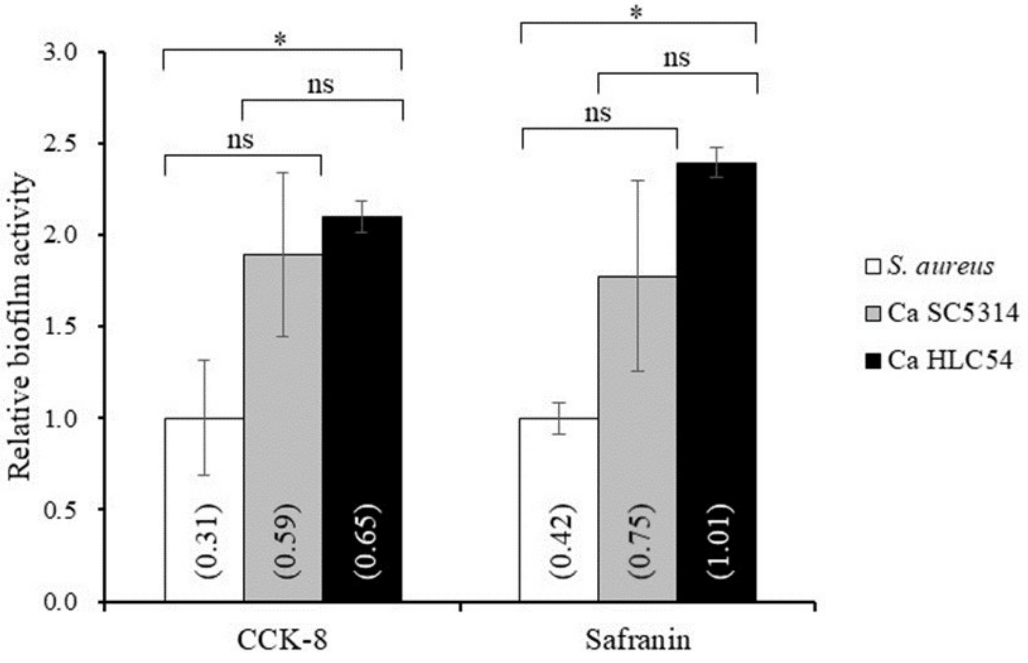

Figure S1. The enhancement of *S. aureus* biofilm using culture supernatants of *C. albicans*. *S. aureus* was seeded on filter membranes to form monomicrobial biofilms over 4 hours and then 0.2-μm filtered supernatants of *S. aureus*, *C. albicans* wild type SC5314 or yeast-locked mutant HLC54 strains that were cultivated for 12h were added into the media separately. Metabolic reduction with CCK-8 or safranin staining assays were used after another 12 hours of incubation and normalized with *S. aureus* supernatant controls. The original mean OD_{450} or OD_{530} values for the assays are shown in parentheses in the columns. * p<0.05, ns = not significant.
